# Evidence of emerging BBB changes in mid‐age apolipoprotein E epsilon‐4 carriers

**DOI:** 10.1002/brb3.2806

**Published:** 2022-11-21

**Authors:** Nourah M. Alruwais, Jenifer M. Rusted, Naji Tabet, Nicholas G. Dowell

**Affiliations:** ^1^ Health science department, College of Applied Studies and Community Services King Saud University Riyadh Saudi Arabia; ^2^ School of Psychology University of Sussex Brighton UK; ^3^ Brighton and Sussex Medical School (BSMS) Brighton UK

**Keywords:** Alzheimer's disease, APOE4, BBB, DCE‐MRI, neuroimaging, structural brain changes

## Abstract

**Introduction:**

Studies have recognized that the loss of the blood–brain barrier (BBB) integrity is a major structural biomarker where neurodegenerative disease potentially begins. Using a combination of high‐quality neuroimaging techniques, we investigated potential subtle differences in BBB permeability in mid‐age healthy people, comparing carriers of the apolipoprotein E epsilon‐4 (APOEε4) genotype, the biggest risk factor for late onset, non‐familial AD (LOAD) with APOEε3 carriers, the population norm.

**Methods:**

Forty‐one cognitively healthy mid‐age participants (42–59) were genotyped and pseudo‐randomly selected to participate in the study by a third party. Blind to genotype, all participants had a structural brain scan acquisition including gadolinium‐based dynamic contrast‐enhanced magnetic resonance imaging acquired using a T1‐weighted 3D vibe sequence. A B1 map and T1 map were acquired as part of the multi‐parametric mapping acquisition.

**Results:**

Non‐significant, but subtle differences in blood–brain barrier permeability were identified between healthy mid‐age APOEε4 and APOEε3 carriers, matched on age, education, and gender.

**Discussion:**

This study demonstrated a tendency toward BBB permeability in APOEε4 participants emerging from mid‐age, with quantitative differences observable on a number of the measures. While the differences did not reach a statistical significance, the results from this study hint at early changes in ε4 carrier BBB that may help identify at‐risk populations and facilitate the development of early interventions to change the trajectory of decline.

## Graphical abstract

This study demonstrated a tendency towards BBB permeability in APOEε4 participants emerging from mid‐age, with quantitative differences observable on a number of the measures. Results from this study may help identify at‐risk populations at an early stage which will be crucial in facilitating the development of early interventions to change the trajectory of decline. To our knowledge, the BBB/APOEε4/healthy mid‐age human studies have not yet been established at the novel age range (45‐59).

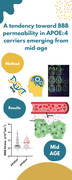

## 1 INTRODUCTION

Alzheimer's disease (AD) is the most common dementia disorder accounting for about 60%–70% of dementia cases (Gourley et al., 1985; World Health Organization, 2022;). As neuronal degeneration is the main feature of AD, the earliest pathophysiological changes behind such defects need to be isolated and identified in at‐risk individuals, in order to deliver a targeted prevention strategy to arrest or delay the disease onset.

Aβ deposition and tau neurofibrillary tangles have been shown to be present in cognitively healthy older adults, which suggests that AD pathology is active years before clinical diagnosis is made (Leal et al., 2018). Recent studies have suggested that AD neuropathology may begin 10–20 years prior to the onset of clinical symptoms (Sperling et al., 2014; Younes et al., 2019).

Both region‐specific and whole‐brain atrophy as well as white matter (WM) tract disruption have been linked to Aβ deposition in patients who are at higher risk of developing late‐onset sporadic AD (LOAD) (Apostolova et al., 2012; Cavedo et al., 2017; Poels et al., 2010; Poliakova et al., 2016).

APOE is a polymorphic protein that has three allelic variants in humans (ε2, ε3, and ε4). The most common APOE allele in populations worldwide is ε3, accounting for 65%–85%, followed by ε4 accounting for 25% of the population, while ε2 is far less common and may even be absent in some ethnic groups (Corbo & Scacchi, 1999; Hubacek et al., 2021; Huebbe & Rimbach, 2017; Mahley, 1988). The APOE gene is implicated in cardiovascular and neurovascular diseases, but the ε4 variant (APOEε4) is independently considered to be the biggest genetic risk factor for LOAD (Corder et al., 1993; Huang & Mucke, 2012). APOEε4 may also influence the rate of cognitive decline most significantly at the early stages (Cosentino et al., 2008; Davignon et al., 1988).

The APOEε4 genotype has effects on multiple aspects of the LOAD chain, such as demyelination, which is thought to contribute to reduced cognitive performance in healthy mid‐age individuals compared with non‐APOEε4 carriers (Bartzokis et al., 2007), lobar micro‐bleeds (MB) causing the neurophysiological function to worsen (Caselli et al., 2009), cortical thinning in regions of the hippocampus contributing to episodic memory decline (Donix et al., 2010), and long‐term memory decline in mid‐age (Taylor et al., 2017). Furthermore, studies have shown that the APOEε4 genotype is a risk factor for developing vascular disease which could promote LOAD via blood–brain barrier (BBB) dysfunction (Montagne et al., 2015, 2016, 2018, 2017, 2020, 2021; Nelson et al., 2016). Therefore, the APOEε4 variant has attracted attention as the single most important known genetic risk factor for non‐familial LOAD.

Evidence suggests that the APOEε4 gene has a different impact on cognition across the lifespan (Han & Bondi, 2008; Lancaster et al., 2020); therefore, age is an important factor in understanding the role and contribution of APOEε4 to AD. Although APOEε4 is associated with an increased occurrence of age‐related cognitive impairment (Deary et al., 2002), unexpectedly, studies show that it could be associated with cognitive advantages in younger population (Dowell et al., 2013; Evans et al., 2013; Rusted et al., 2013; Taylor et al., 2017). Even in healthy mid‐age adults, the APOEε4 variant was not associated with significant cognitive change (Cacciaglia et al., 2018) but may be the point of transition toward poorer performance, being associated with steeper age‐related decline in cognitive ability (Cacciaglia et al., 2018; Caselli et al., 2009; Lancaster et al., 2017, 2020; Mishra & Brinton, 2018).

Brain blood vessels provide a strong structural framework for the delicate brain. An important component of the cerebrovascular structure is the BBB which is a semi‐permeable membrane that prevents toxins and pathogens, including blood‐derived materials, from entering the brain (Keaney & Campbell, 2015). In AD, the BBB is suspected of being dysfunctional, allowing leakage into the brain tissue, which in turn leads to neuronal damage and accumulation of neurotoxins (Nelson et al., 2016; Zenaro et al., 2017). A leaky BBB may represent one of the first stages leading to neuronal damage contributing to cognitive decline in neurodegenerative disease (Montagne et al., 2015; Nelson et al., 2016; Sweeney et al., 2018). Dynamic contrast‐enhanced magnetic resonance imaging (DCE‐MRI) has been used together with the Patlak fitting model to measure BBB leakage as well as localization of the leakage (Barnes et al., 2016; Montagne et al., 2015, 2020; Thrippleton et al., 2019; Tofts, 2010; Tofts & Kermode, 1991). The Patlak model is able to quantify several parameters including K*trans* and Vp. Vp is the fractional plasma volume in the selected region, while K*trans* is the rate at which the contrast agent in the blood plasma passes from the BBB to the extracellular extravascular space. Consequently, K*trans* is a good proxy of BBB permeability (Tofts, 2010).

The rate of BBB breakdown has been shown to increase with age, both in people with LOAD and also in healthy older individuals, which indicates that BBB disruption is part of normal ageing (Verheggen et al., 2020). BBB leakage was also detectable prior to the development of measurable cognitive decline at a preclinical stage in LOAD (Iturria‐Medina et al., 2016). Understanding the timing of BBB breakdown is critical for prevention and feasible interventions. To date, most BBB studies have been on murine models (Bell et al., 2012; Daneman et al., 2010) or human postmortem individuals (Sengillo et al., 2013; Toledo et al., 2013), but in a seminal study, Montagne et al. (2015) reported findings from a cohort of non‐cognitively impaired (NCI) and mild cognitively impaired (MCI) participants of a wide age range (23–91), categorized by age: young NCI, older NCI, and MCI groups. The study explored two indices of BBB integrity, namely in vivo K*trans* BBB measurements and CSF/plasma albumin ratio (Qalb). Their findings indicated age‐related changes in NCI participants that suggest BBB breakdown is part of normal aging, and BBB leakage was found to be higher in age‐matched MCI patients, which suggests BBB breakdown contributes to early cognitive impairment. The loss of BBB integrity increased particularly in the hippocampus, a region identified as displaying earliest and most extensive damage in LOAD postmortem cases (Sengillo et al., 2013), suggesting the target region of early BBB breakdown in LOAD. A more recent study on vascular MCI (vMCI) participants found that BBB leakage increased in vMCI patients compared to healthy age‐matched controls and that cognitive decline significantly correlated with the rate of BBB leakage in vMCI patients (Li et al., 2021). A further study on 245 participants, mean age 67.3, revealed that the presence of APOEε4 genotype contributes to BBB breakdown in both cognitively healthy carriers and MCI patients (Montagne et al., 2020). BBB breakdown increased in healthy APOEε4 relative to non‐carriers as indicated by K*trans* levels, and correlated with the increase in two inflammatory markers: Cyclophilin A and matrix metalloproteinase‐9. The CypA‐MMP‐9 pathway is a brain pro‐inflammatory pathway in pericytes^1^ of the BBB endothelial walls (Bell et al., 2012). BBB breakdown was found to be higher in APOEε4/MCI individuals compared to APOEε4/healthy, and the increased BBB permeability was identified in the hippocampal and medial temporal lobe regions (Montagne et al., 2020). These findings are consistent with a human postmortem brain tissue study in LOAD APOEε4 carriers which suggested that BBB breakdown is initiated by pericyte degeneration, in turn due to the accumulation of the pro‐inflammatory cytokines CypA and MMP‐9 in the pericytes and endothelial cells (Halliday et al., 2016). The age at which BBB leakage begins is of great importance. A DCE‐MRI study involving people in the early stages of LOAD (age range: 59–85) found that BBB dysfunction was significantly increased in several regions of white and grey matter in these individuals compared to healthy age‐matched controls and that cognitive decline increased significantly with the increase in the rate of BBB leakage (van de Haar et al., 2016, 2017). However, BBB leakage is increased in older adults compared to mid‐age adults (Verheggen et al., 2020) and increased in middle aged rats (∼50 years old in human age) compared to young rats (Bors et al., 2018) suggesting, as reported by Montagne et al. (2018), that BBB dysfunction is part of normal aging.

To summarize:

1. BBB breakdown is thought to be one of the first structural changes leading to LOAD starting somewhere around mid‐age.
2. BBB leakage is found to be higher in APOEε4 carriers compared to non‐carriers in both LOAD and in healthy older population.
3. The hippocampus is a region identified to show the earliest changes in LOAD and higher in APOEε4 carriers compared to non‐carriers.

Therefore, understanding from where and how APOEε4 may be implicated in the trajectory of pathological changes leading to LOAD is crucial in tackling this neurological disease, especially when LOAD diagnosis is often delayed until there are observable changes in cognitive performance. As structural imaging studies on mid‐age APOEε4 population are often contradictory, and BBB/APOEε4/healthy mid‐age human studies have not been yet established, we hypothesized that very subtle BBB differences between APOEε4 and APOEε3 carriers may be identified in our narrow age range, identified here as mid‐age.

The aim of this study was to investigate the early detection of subtle BBB changes in people at a higher risk of developing late‐onset, non‐familial AD later in life (APOEε4 carriers) and to identify differences between APOEε3 and APOEε4 in our novel mid‐age range where these very subtle changes may start to be detectable through DCE‐MRI. The study aims to increase our knowledge of the potential neuropathological trajectory in mid‐age individuals who carry the additional risk factor of the APOEε4 genotype. Specifically, we aim to assess whether APOEε4 is associated with subtle BBB leakage in mid‐age. This may provide the opportunity for early intervention to maintain and improve individuals’ vascular, and therefore cognitive, health as they age.

## 2 METHODS

### 2.1 Participants and genotyping

Forty‐one healthy mid‐age participants (age, 52 ± 4 years; range, 42–59 years; 33 females, 8 males) participated in the study. Participants were excluded if they were non‐Caucasian (due to ethnic differences in physiological consequences of APOE gene variants) (Farrer et al., 1997); they had a current physical and/or mental illness (self‐reported); they had any MRI contraindications such as implantable devices (e.g., non‐MRI compliant cardiac pacemaker, metal fragment lodged in the eyes or body, large or dark tattoos on the head or neck, pregnancy or claustrophobia); and they had any of the contrast media contraindications (including asthma, history of renal disease/kidney problems, allergies/sensitivity to contrast media). Participants were considered eligible for the imaging phase when preliminary blood test results showed normal kidney function indicated by an estimated glomerular filtration rate >60. The study protocol was approved by The Brighton and Sussex Medical School Research Governance and Ethics Committee, and DNA samples were collected using buccal swabs and analyzed for each participant for APOE status by Bioresearch Technologies LGC Hoddesdon UK. Only APOEε4 and APOEε3 carriers were invited to participate through a triangulated anonymized selection completed by a third party. Participants were consented at the beginning of each study phase. One participant withdrew prior to the imaging phase, citing claustrophobia. The groups taking part in the final imaging phase included 20 APOEε4 carriers (*n* = 4 ε4/ε4 and *n* = 16 ε4/ε3) and 20 APOEε3 carriers (all ε3/ε3). After the start of the scanning session, one participant withdrew from the DCE‐MRI phase and three BBB imaging data were lost due to substantial image artifacts, and the final BBB data processed were from 36 participants (*n* = 17 APOEε4, *n* = 19 APOEε3). The sample size was computed using nQuery power calculator

(https://www.statsols.com/nquery) and was motivated by prior research (Cramer et al., 2014; Dowell et al., 2016; Montagne et al., 2016).

### 2.2 BBB imaging protocol

All imaging data were acquired on a Siemens 3 Tesla MRI scanner and 32‐channel phased‐array, receive‐only, head coil. The DCE‐MRI data were acquired using a T1‐weighted 3D vibe sequence with TR = 2.56 ms, TE = 0.86 ms, flip angle = 15°, GRAPPA with parallel imaging factor = 2, acquired matrix = 96 × 72 × 28, reconstructed into 36 sagittal slices of 5.0 mm slice thickness, field of view = 240 × 240 × 180 mm^3^. The DCE acquisition comprised 432 repeated volumes, each with an acquisition time of 2.4 s, resulting in a total measurement time of approximately 17 min. The infusion of contrast agent was initiated after 10 T1w VIBE volumes were collected to provide a baseline image intensity measure. Gadoterate meglumine (Dotarem) was administered remotely using an automated injector with speed of 3 ml/s, followed by a saline flush at the same rate. A single dose of the contrast agent Doterem was injected based on participant weight (0.05 mmol/kg body weight). Signal drift during DCE‐MR acquisition will lead to a poor estimate of BBB leakage (K*trans*) (Vos et al., 2016). To estimate signal drift during the DCE acquisition, three phantoms consisting of nickel‐doped agarose gel in 50 cm^3^ test‐tubes (Diagnostic Sonar Eurospin Gels, Diagnostic Sonar Ltd., UK) were placed within in prescribed the field‐of‐view. The T1 and T2 values were chosen to lie within physiological range and were placed on the outside of the head coil to minimize heating (and the concomitant T1 change) from the participant. A B1 map and a T1 map were acquired as part of the multi‐parametric mapping acquisition, obtained in the same scanning session. The B1 map was used in the DCE analysis to minimize the corruptive effects of subtle long‐term movement on K*trans* (Sacolick et al., 2010).

### 2.3 Image analysis

B1 maps, T1 maps, and the DCE‐MRI volumes were all co‐registered to a common participant image space, using SPM12. A priori regions‐of‐interest (ROIs) were selected from the MNI‐space atlas and co‐registered to participant space for region‐specific statistical analysis. In‐house software, written in the C programming language by one of our research team (NGD), was used to obtain K*trans* and Vp in a pixel wise manner by fitting the DCE‐MRI data, T1 map and B1 map, using the Patlak model. A B1 map was used to mitigate the spatially dependent signal variation across the DCE images as a result of the inhomogeneous receive sensitivity profile. Since K*trans* is derived from the pixel‐wise signal enhancement observed during the entire DCE acquisition, a B1 correction should improve the reliability of K*trans* in the presence of subtle long‐term movement (Sacolick et al., 2010). Smoothing (Gaussian kernel 4‐mm isotropic) was applied to the T1 maps and DCE‐MRI data to improve the robustness of the fit. On the basis of previous literature, six ROIs were selected for the BBB analysis in both the right and left hemispheres: hippocampus, white matter anterior cingulate cortex (WM ACC), anterior cortex, and subcortical probe of the hippocampus, as well as anterior and posterior parahippocampal gyrus. The ROIs are located in the frontal and temporal lobes (cortical and subcortical regions) including different tissue types (GM and WM), selected to cover a variety of brain regions associated with different patho‐physiological changes in the early clinical stages of LOAD (Bobinski et al., 1999; Kantarci et al., 2017; Smith, 2002; Thompson et al., 2008) and also in healthy middle‐aged APOEε4 carriers (Cacciaglia et al., 2018, 2019; Donix et al., 2010; Klunk et al., 2007; Liu et al., 2010; Mishra & Brinton, 2018; Operto et al., 2018; Slattery et al., 2017; ten Kate et al., 2016). A histogram analysis of each ROI was performed to identify subtle diffuse differences in K*trans* values between genotype groups. As mentioned earlier, K*trans* is used as a proxy for BBB permeability. Two features of the histograms are considered here: normalized peak height and peak position. Peak height is sensitive to the homogeneity observed for the parameter. Subtle and diffuse damage would lead to increased variance in K*trans* resulting in a broader histogram peak with reduced height after normalization. Histogram position (the distribution mode) is sensitive to a more global shift in K*trans*, that is, increase BBB permeability, perhaps due to pathological changes, is identified by increase in peak position and decrease in peak height (Tofts et al., 2003). Measurement of normalized peak height and peak position was obtained for each participant and a one‐way analysis of covariance (SPSS version 26) was used to identify differences between APOEε3 and APOEε4 groups in the selected ROIs. To account for differences in size of the ROIs, the total number of pixels (i.e., volume) of each ROI was added as covariates in the statistical model. Outlier was identified and removed using ROUT test in (GraphPad Prism 9.1.1), and BBB K*trans* values were also compared using violin plots generated in (GraphPad Prism 9.1.1) to better visualize data distribution and differences between groups. This study generated two comparisons across 10 different ROIs, and since this study has a single hypothesis being tested, statistical significance was retained at *p* < 0.05 following previous studies (Bors et al., 2018; Li et al., 2021; Verheggen et al., 2020). Effect size was also calculated using Cohen's *d* test using the means and standard deviations of both groups.

## 3 RESULTS

**FIGURE 1:**
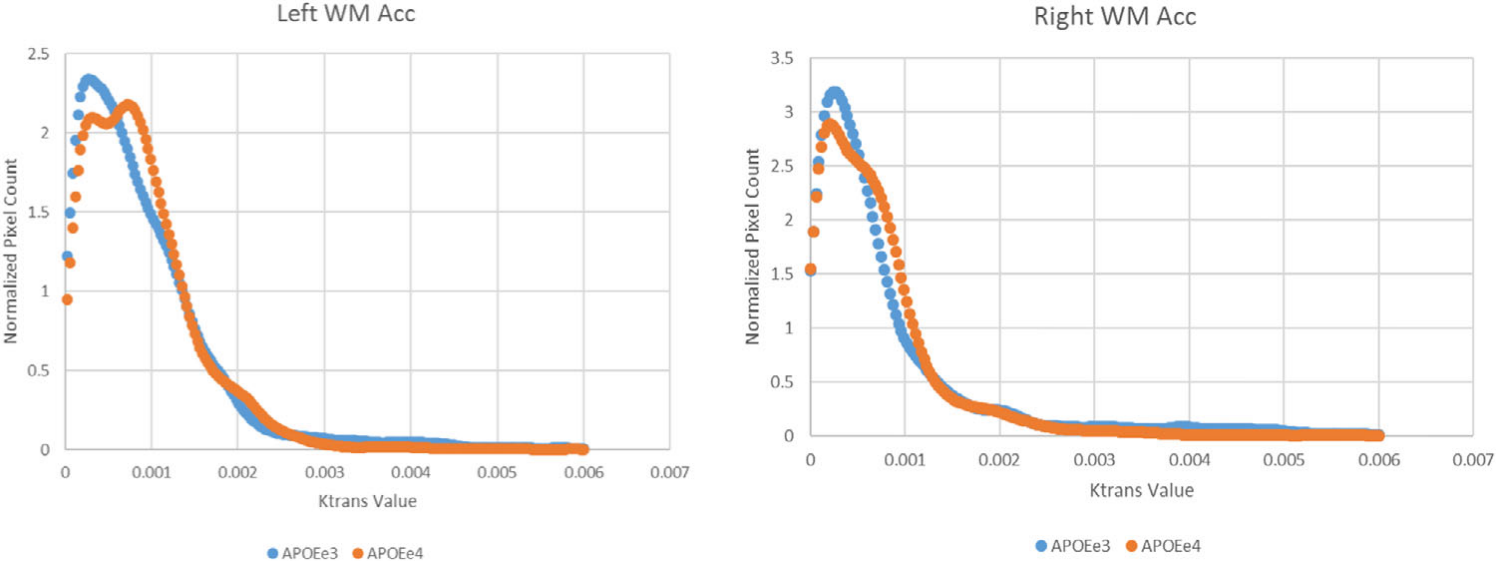
Normalized blood–brain barrier (BBB) histograms of the left and right white matter anterior cingulate cortex. APOEe3 group is shown in blue, and APOEe4 group is shown in red.

**FIGURE 2:**
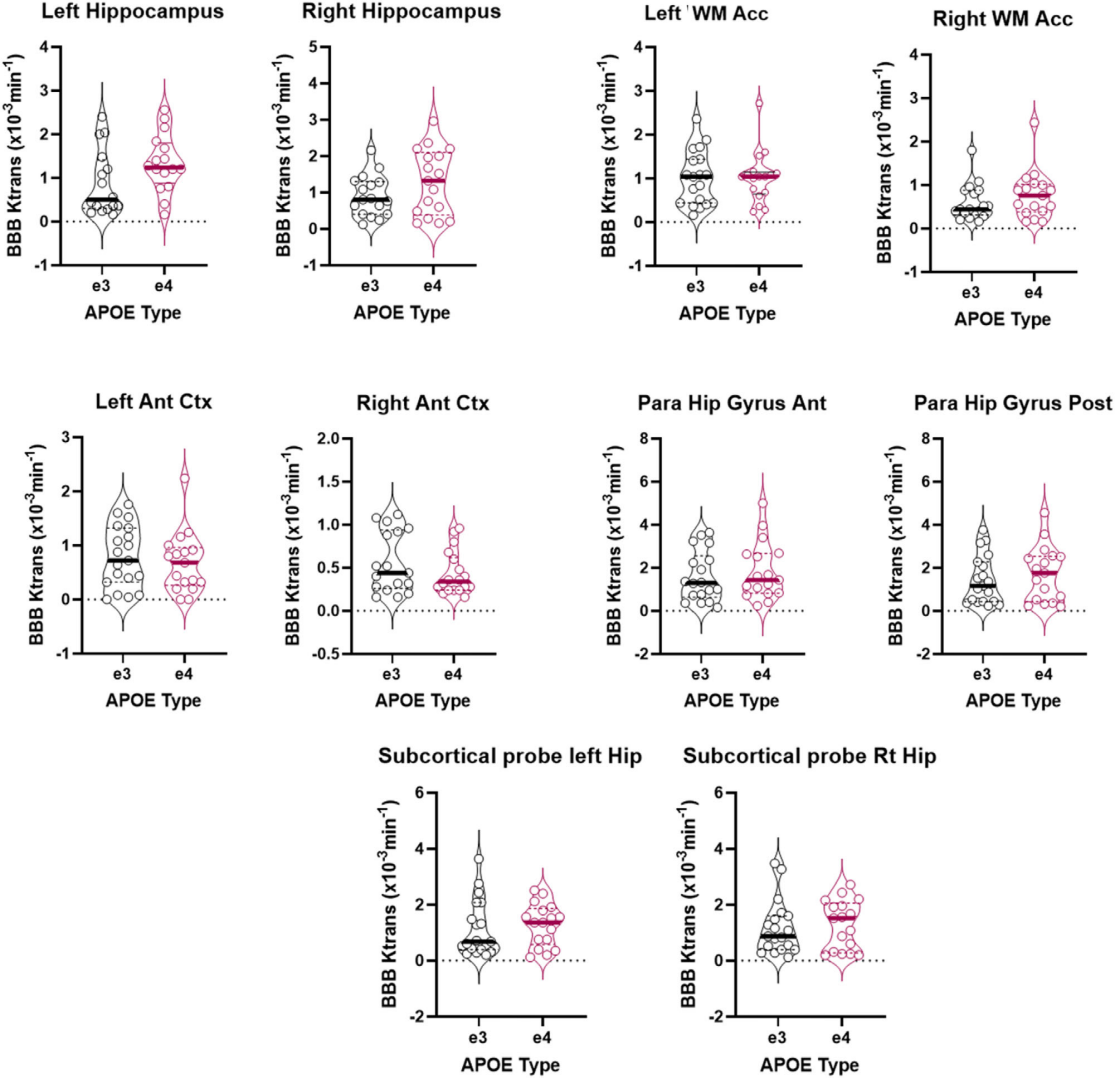
Violin plots showing mean regional blood–brain barrier (BBB) K*trans* in 10 brain regions in APOEε3 (black, *n* = 19) and APOEε4 (red, *n* = 17) carriers. Group median is shown as solid horizontal line. No significant difference was found by analysis of covariance (ANCOVA) (mean difference). Regions: left hippocampus, right hippocampus, left white matter anterior cingulate cortex, right white matter anterior cingulate cortex, left anterior cortex, right anterior cortex, anterior parahippocampal gyrus, posterior parahippocampal gyrus, left hippocampus subcortical probe, and right hippocampus subcortical probe

**FIGURE 3:**
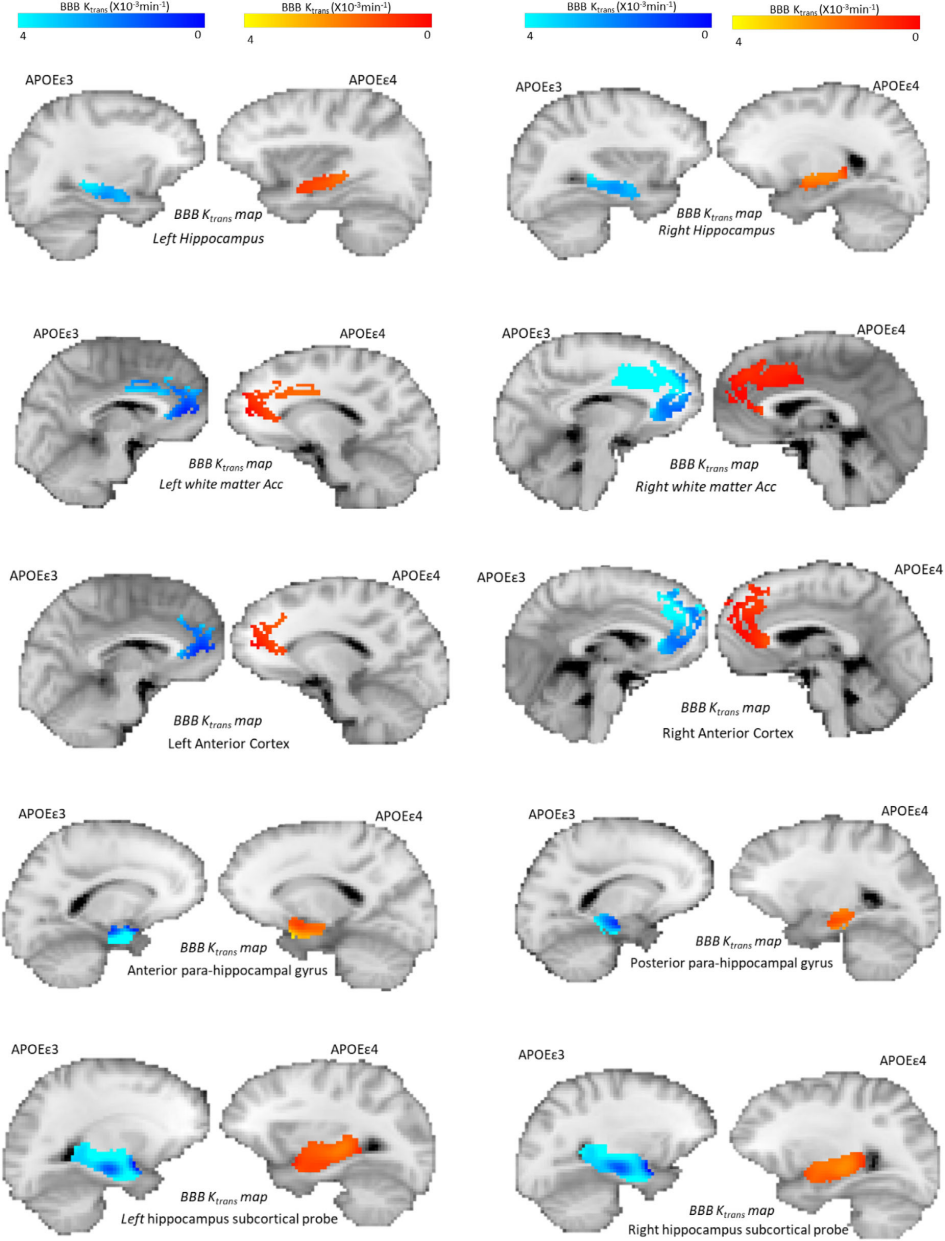
Representative K*trans* maps in 10 brain regions from individual APOEε3 (dark/light blue, *n* = 19) and APOEε4 (red/yellow, *n* = 17) carriers. The maps are overlaid on T1‐weighted contrast images in the native space of the participants. Regions: left hippocampus, right hippocampus, left white matter anterior cingulate cortex, right white matter anterior cingulate cortex, left anterior cortex, right anterior cortex, anterior parahippocampal gyrus, posterior parahippocampal gyrus, left hippocampus subcortical probe, and right hippocampus subcortical probe. The regions‐of‐interest (ROIs) and brain images were taken from individual participants carrying either APOE ε3 or APOEε4 genotype.

BBB K*trans* histograms of 36 healthy mid‐age (45–59) participants were generated showing very subtle but lower peak height and higher peak position in the APOEε4 group in most ROIs. Right and left WM Acc histograms are shown in Figure 1. BBB permeability measured by BBB K*trans* showed no significant genotype‐dependent differences in any of the selected ROIs (Table 1). It is interesting to mention, however, there were very subtle (non‐significant) directional differences in favor of the APOEε4 group showing increased K*trans* (greater BBB permeability) in eight out of the possible 10 comparisons made in the subcortical regions shown in the violin plots (Figure 2). These differences were not seen in the cortical ROIs. Additionally, small effect size was observed in several regions indicating very subtle group differences. Moreover, BBB K*trans* histograms were generated showing lower peak height and higher peak position in the APOEε4 group in all ROIs (Figure 2). We also present representative K*trans* maps from two participants (one from each genotype group) for our chosen ROIs (Figure 3).

**TABLE 1:** Mean (standard deviation) and significance level of blood–brain barrier (BBB) K*trans* histogram peak height and peak position for each region‐of‐interest (ROI) for APOEe3 and APOEe4 carriers

	APOEe3(19)	APOEe4(17)	p	d	APOEe3(19)	APOEe4(17)	p	d
	Peak	Height			Peak Position			
Left hippocampus	4.66 (1.8)	4.41 (1.5)	.704	0.15	1.21 (1.2)	1.35 (0.7)	.791	0.14
Right hippocampus	3.65 (2.7)	4.06 (4.4)	.741	0.11	1.16 (0.9)	1.29 (0.9)	.738	0.14
Left WM ACC	4.00 (2.3)	3.43 (1.7)	.535	0.28*	1.00 (0.6)	1.02 (0.6)	.789	0.03
Right WM ACC	3.98 (1.8)	4.16 (2.1)	.468	0.09	0.77 (0.9)	0.78 (0.6)	.629	0.01
Left anterior cortex	9.95 (9.9)	7.41 (3.1)	.442	0.35*	0.78 (0.6)	0.68 (0.6)	.302	0.16
Right anterior cortex	4.37 (1.6)	4.20 (2.2)	.922	0.09	0.52 (0.5)	0.56 (0.4)	.901	0.09
Parahippocampal gyrus anterior	2.43 (2.0)	2.11 (2.0)	.648	0.16	1.90 (1.7)	1.85 (1.3)	.951	0.03
Parahippocampal gyrus posterior	2.78 (1.3)	3.20 (1.4)	.427	0.31*	1.44 (1.1)	1.72 (1.3)	.251	0.23*
Subcortical probe Left hippocampus	3.44 (1.5)	3.39 (1.9)	.974	0.03	1.16 (1.0)	1.29 (0.8)	.646	0.14
Subcortical probe right hippocampus	3.55 (1.6)	3.56 (1.1)	.842	0.01	1.18 (1.0)	1.29 (0.9)	.389	0.12

*Note*: Peak position is in unit (×10^−3^ min^−1^).

Abbreviations: d, Cohen's effect size; WM ACC, white matter anterior cingulate cortex.

Significant (p < .05).

* Small effect size detected.

## 4 DISCUSSION

The necessity to tackle AD at its earliest stages has been increasingly acknowledged in the past few years. Early prevention and intervention are assisted by a better understanding of the early biological changes that occur in the human body prior to the development of measurable cognitive decline. Here, we recognize the strongest known genetic risk factor for LOAD (APOEε4) and investigate its effect on mid‐age individuals who have no apparent symptoms of cognitive decline, comparing them to the more common APOE genotype (APOEε3). This study has served to illuminate some underexplored areas in the neuropathological pathway, and we are able to answer some specific research questions from this study.

BBB breakdown is thought to be one of the initiation points where many neurological diseases begin. BBB breakdown is known to lead to AD and has been identified in carriers of the APOEε4 gene with early onset AD (van de Haar et al., 2016, 2017). In this study, we compared BBB permeability in age‐matched APOEε4 and APOEε3 carriers (all participants being mid‐age and disease free), by measuring contrast (Gd) leakage across the BBB at regions identified in the literature to be dysfunctional in early AD. A directional difference was observed in subcortical regions including the hippocampus toward higher BBB leakage in APOEε4 carriers. This is consistent with several studies that have identified BBB breakdown in the hippocampus to be the first region affected as part of aging, in AD and in APOEε4/MCI carriers (Montagne et al., 2015, 2020; Sengillo et al., 2013; Verheggen et al., 2020). Although these trends are not statistically significant in our present cohort, these results should be considered with findings from our same research group that identified structural differences in mid‐age between APOEε3 and APOEε4 (Dowell et al., 2016). In that context, the findings presented here are in line with the hypothesis that gradual and very subtle change in the BBB tight junction begins in APOEε4 carriers at mid‐age in subcortical regions before it progresses to other regions, even in healthy individuals who show no cognitive changes. The findings here are directly in line with previous AD studies that suggest BBB breakdown begins prior to the development of any cognitive defects and as early as mid‐age (Hussain et al., 2021; Iturria‐Medina et al., 2016). Iturria‐Medina et al. (2016) investigated vascular integrity on a large late‐onset AD cohort mean age 73.4 (SD 7.3) and suggested that early vascular dysfunction is associated with early cognitive decline. Bors et al. (2018) found an age‐related BBB breakdown when comparing between cognitively unimpaired young and mid‐age rats. To our knowledge, no human studies have previously investigated BBB integrity exclusively in cognitively healthy mid‐aged APOEε4 carriers, strictly at the 45–59 age range. Our observation of the very early signs of BBB disruption occurring in the cognitively healthy mid‐age individuals may be considered a novel and important finding. Perhaps, the selected mid‐age range in this study is slightly younger for measurable BBB changes using non‐invasive DCE‐MRI, and a slightly older mid‐age range 55–65 rather, may detect more significant BBB permeability/genetic differences in healthy population. A longitudinal study on the same cohort in (±5 years) and a replicate of the same study on a larger sample may ascertain these directional differences.

**FIGURE 4:**
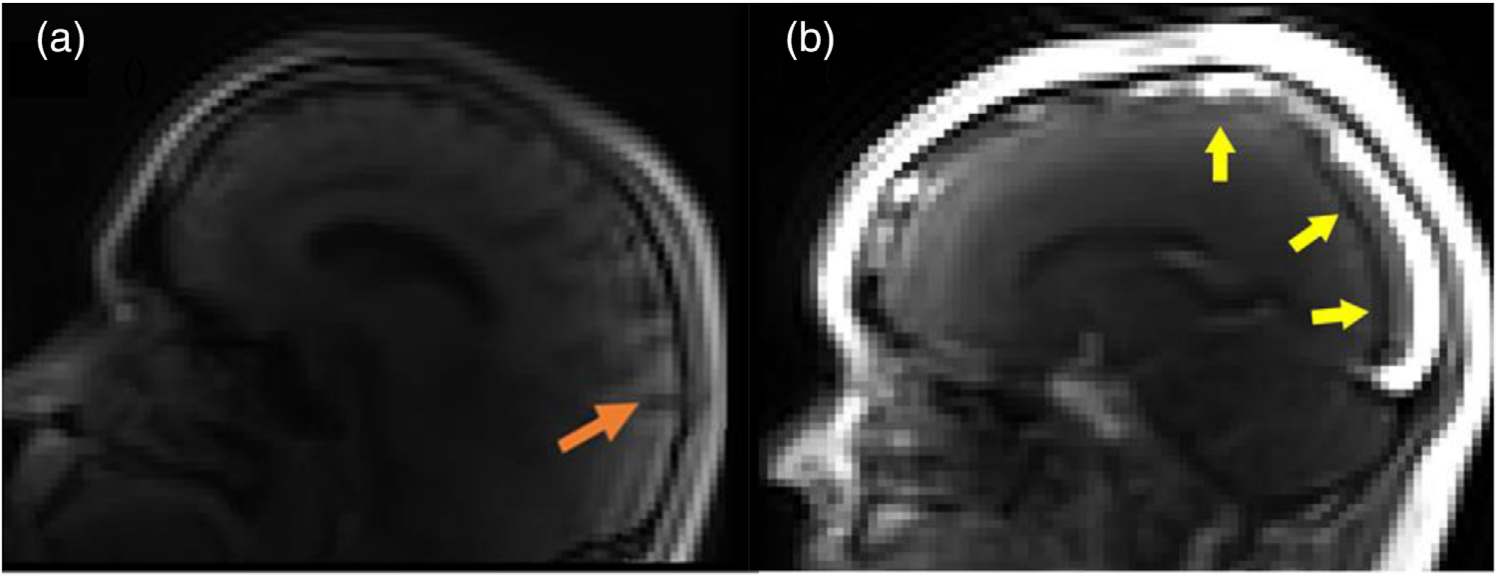
(a) Image of a participant brain sagittal view, showing motion artifacts due to movement during the scan and (b) sagittal view showing the effect of Gd contrast brightness on image quality

Although comparable to other structural imaging and DCE‐MRI studies, an acknowledged limitation of our study is the small sample size which may limit the generalization of results. The length of scanning time was both an obstacle for some participants due to discomfort, and produced significant image artifacts due to participants’ movement throughout the scanning session. This was quantified in the realignment phase during image analysis, but the effects were not completely resolved (Figure 4a). Additionally, artifacts and image quality were impacted by the increased brightness from the high concentration of Gd after bolus injection (Figure 4b). During bolus injection, Gd is highly concentrated in the vessels, and the increased brightness results in image artifact across a substantial region of the image, which was in about 50% of the volumes acquired in DCE‐MRI. Finally, differences in ROI sizes, the selection of small anatomical structures for the ROIs, and subtle head movement during the scan resulted in failure of the Patlak model fitting, especially in cortical regions. This may be addressed in future studies by (1) selecting larger ROIs to improve signal‐to‐noise ratio and (2) the (17‐min) DCE‐MRI is the last part of a longer scanning session in this study which was about 70 min, and motion artifact maybe addressed in future work by shortening the overall scanning session or separating the DCE‐MRI from the initial structural imaging. This may improve participant comfort and reduce motion artifacts. In addition, the artifacts from the brightness caused by the high Gd concentrations in vessels could be tackled with further optimization of the DCE pulse sequence acquisition parameters.

## 5 CONCLUSION

In conclusion, using the less invasive DCE‐MRI approach, the increasingly preferred methodology for BBB leakage measurements (Verheggen et al., 2020; Wong et al., 2017) rather than the highly invasive method using lumber puncture, we completed the first BBB permeability study on cognitively healthy, mid‐aged APOEε4 and APOEε3 carriers. As subcortical/hippocampal regions are the first identified regions to be affected in early AD (Montagne et al., 2015; Nation et al., 2019), although non‐statistically significant, our BBB K*trans* data suggest that the subcortical regions including the hippocampus consistently show emerging differences, and here showing subtly higher BBB permeability in the direction of APOEε4 carriers. These differences are detectable using DCE‐MRI imaging as early as the mid‐forties, and prior to the development of any cognitive changes. A longitudinal follow‐up on the same cohort would be recommended to further analyze the development of the subtle differences identified between the groups in the present work.

## CONFLICT OF INTEREST

The authors declare no conflict of interest.

### PEER REVIEW

The peer review history for this article is available at https://publons.com/publon/10.1002/brb3.2806

## Data Availability

The anonymized data that support the findings of this study are available on request from the corresponding author. To be compliant with our ethics, the data are not publicly available.

## NOTE

1 Pericytes are cells along vessel walls which are extremely important in CNS vessels as they have a major role in maintaining the BBB integrity. Pericytes degeneration is found in AD (Sengillo et al., 2013).
